# The Interplay of Mitophagy and Inflammation in Duchenne Muscular Dystrophy

**DOI:** 10.3390/life11070648

**Published:** 2021-07-04

**Authors:** Andrea L. Reid, Matthew S. Alexander

**Affiliations:** 1 Department of Pediatrics, Division of Neurology at Children’s of Alabama, University of Alabama at Birmingham, Birmingham, AL 35294, USA; areid01@uab.edu; 2 UAB Center for Exercise Medicine (UCEM), University of Alabama at Birmingham, Birmingham, AL 35205, USA; 3 Department of Genetics, University of Alabama at Birmingham, Birmingham, AL 35294, USA; 4 UAB Civitan International Research Center (CIRC), University of Alabama at Birmingham, Birmingham, AL 35233, USA

**Keywords:** mitophagy, DMD, inflammation, dystrophin, dystrophy

## Abstract

Duchenne muscular dystrophy (DMD) is an X-linked neuromuscular disease caused by a pathogenic disruption of the *DYSTROPHIN* gene that results in non-functional dystrophin protein. DMD patients experience loss of ambulation, cardiac arrhythmia, metabolic syndrome, and respiratory failure. At the molecular level, the lack of dystrophin in the muscle results in myofiber death, fibrotic infiltration, and mitochondrial dysfunction. There is no cure for DMD, although dystrophin-replacement gene therapies and exon-skipping approaches are being pursued in clinical trials. Mitochondrial dysfunction is one of the first cellular changes seen in DMD myofibers, occurring prior to muscle disease onset and progresses with disease severity. This is seen by reduced mitochondrial function, abnormal mitochondrial morphology and impaired mitophagy (degradation of damaged mitochondria). Dysfunctional mitochondria release high levels of reactive oxygen species (ROS), which can activate pro-inflammatory pathways such as IL-1β and IL-6. Impaired mitophagy in DMD results in increased inflammation and further aggravates disease pathology, evidenced by increased muscle damage and increased fibrosis. This review will focus on the critical interplay between mitophagy and inflammation in Duchenne muscular dystrophy as a pathological mechanism, as well as describe both candidate and established therapeutic targets that regulate these pathways.

## 1. Introduction

### 1.1. Duchenne Muscular Dystrophy

Duchenne muscular dystrophy (DMD) is a severe and progressive neuromuscular disease that affects 1:5000 live male births, making it the most common form of muscular dystrophy [1,2,3]. DMD is caused by a disruption of the *DYSTROPHIN* gene that results in the loss of or production of a non-functional dystrophin protein [2,4]. Lack of dystrophin in the muscle results in myofiber death and fibrosis, which induces fatal cardiac arrhythmia and/or respiratory failure in DMD patients [5,6]. Dystrophin-replacement approaches via exon-skipping or micro-dystrophin gene therapies have been showing promising results in the prevention of muscle necrosis (recently reviewed by Sun et al. [7]); however, they do not fully rescue all pathological outcomes [8,9]. Moreover, these exon-skipping therapies are effective only in patients with specific pathogenic *DYSTORPHIN* variants, and thus only a subset of DMD patients are amenable to these strategies [7,10]. A combinatorial approach to DMD treatment strategy is the most likely to succeed, and thus it is important to understand the driving pathological mechanisms at play in DMD.

The loss of dystrophin at a cellular level has been well characterized [11,12]. Briefly, dystrophin is an integral protein at the sarcolemma where it connects the extracellular matrix proteins with the intracellular cytoskeleton, allowing for muscle force transduction across myofibers [13]. Without dystrophin to act as a ‘shock absorber’ to stabilize the myofiber membrane, muscle contractions create tears in the muscle membrane [14]. Calcium ions are able to freely enter the muscle cell rather than through normal channel-mediated regulation, and that results in the activation of a multitude of cascades that induce muscle damage. In parallel, muscle satellite cells attempt to regenerate injured muscle and become depleted and exhausted due to membrane fragility caused by the lack of dystrophin, subsequently leading to impaired muscle regeneration and myogenesis [15]. At a physiological level, this results in muscle necrosis and the infiltration of fat and immune cells into the muscle, which diminishes muscle capacity and function [16]. Two additional key aspects to the DMD pathology are the high levels of chronic inflammation and impaired mitophagy and the clearance of defective mitochondria. In this review, we will discuss the impact of mitophagy on inflammation in DMD, as well as therapeutic approaches that target this pathway. This combinatorial approach to DMD treatment might be used in tandem with gene therapy to treat the systemic issues associated with dystrophic disease pathologies. 

### 1.2. Chronic Inflammation in DMD

Under normal conditions, inflammation is necessary for tissue recovery after injury. However, in DMD where there is chronic injury due to the unstable muscle membrane, inflammation becomes chronic and over-activated throughout the entire muscle [17]. It begins a vicious series of events, where inflammation stimulates further pro-inflammatory cytokine signals, leading to immune infiltration into the muscle and creating a fibrotic and rigid muscle environment. It has already been well established that chronic inflammation in DMD drives disease pathology [18]. Currently, the standard DMD treatment is corticosteroids as it is anti-inflammatory and helps patients maintain muscle strength and extends life expectancy with higher efficacy the earlier the steroid regiment is given to the patient [19,20,21,22,23,24,25]. However, long-term use of corticosteroids does have detrimental side effects, including excessive weight gain, hypertension, glucose intolerance, increased bone fraction risk, and mood modulation [21,26,27]. Thus, elucidating other anti-inflammatory treatment options are essential for DMD patients.

There are two main inflammatory pathways that have been shown to be increased in expression levels in DMD. The first is the NFκB pathway, where TNFα stimulates the transcription factor complex NFκB, causing it to translocate into the nucleus and promote the transcription of pro-inflammatory cytokines [28,29]. In dystrophic muscle, both TNFα and NFκB are significantly upregulated [30,31]. There have been numerous studies showing that different approaches to inhibiting NFκB have been effective in reducing inflammation and attenuating dystrophic muscle pathology, such heterozygous deletion of p65 (NFκB) and pharmacological inhibition of IKK [32,33]. There are several types of small molecules that inhibit NFκB that are in clinical trials for muscular dystrophies as well as other diseases with chronic inflammation characteristics (recently reviewed by Ramadass et al. [34]).

**Figure 1:**
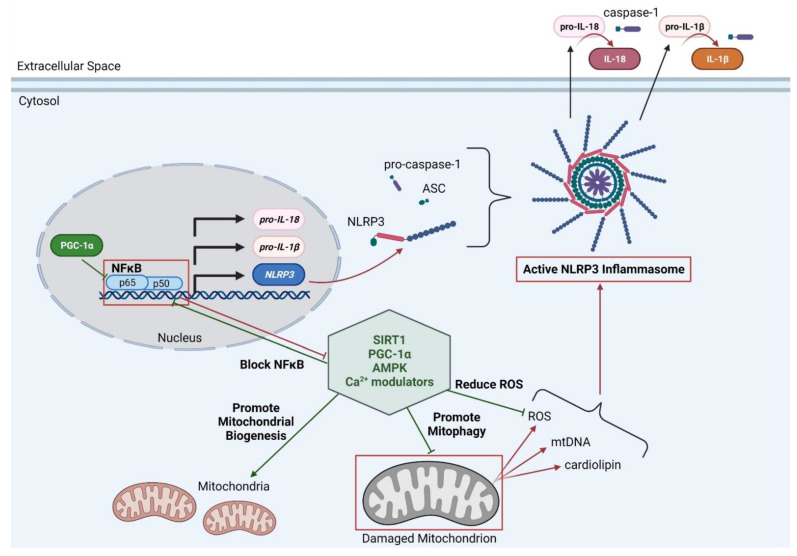
Schematic of the relationship between mitochondria and inflammation. NFκB and NLRP3 inflammasomes are major sources of inflammation in DMD as they activate pro-inflammatory cytokines. Damaged mitochondria can activate these inflammatory mechanisms by its excessive release of reactive oxygen species (ROS), mitochondrial DNA (mtDNA), and cardiolipin. Studies have shown that mitophagy is impaired in DMD, which contributes to the chronic inflammation as activated by defective mitochondria. Accumulating evidence suggests that improving mitophagy and/or targeting the secondary cascades caused by defective mitochondria (e.g., ROS), will lead to reduced inflammation and an overall increase in dystrophic muscle pathology. Highlighted in the center are some key modulators that have been shown affect mitophagy, ROS-mediated cascades, and inflammation, thus representing therapeutic targets for DMD.

The other main inflammatory pathway is the nucleotide-binding oligomerization domain (NOD)-like receptor family pyrin domain containing 3 (NLRP3) inflammasome pathway. NLRP3 inflammasome becomes functional in two stages—first the priming stage, and subsequently the activation stage. The NLRP3 inflammasome must be primed before it can be activated and NFκB activation can act as the primer for NLRP3 by promoting the transcription of some of the NLRP3 inflammasome components and pro-inflammatory cytokines, including *NLRP3*, *pro-IL-IB* and *pro-IL-18*. In the cytosol, NLRP3 binds to apoptosis-associated speck-like protein containing a caspase-activation and recruitment domain (CARD), a domain (ASC) and pro-caspase-1, forming the active inflammasome [35]. Self-activation of pro-caspase-1 cleaves itself to the active form, caspase-1. Caspase-1 subsequently cleaves the pro-inflammatory cytokines to its active state, resulting in high levels of active IL-1β and IL-18 [36] (Figure 1). The NLRP3 inflammasome is significantly upregulated in *mdx* mouse muscle, both at the mRNA and protein levels (4-fold and 3-fold, respectively) [37], which is consistent with the increased NFκB expression observed in DMD pathology. Interestingly, there is accumulating evidence that mitochondria may be a critical regulator of NLRP3 inflammasome-mediated inflammation. 

### 1.3. Impaired Mitophagy in DMD

Numerous studies have demonstrated that mitochondrial dysfunction is one of the first characteristics that can be seen in dystrophic muscle before the overt breakdown of muscle, suggesting that it could be a significant contributor to the pathology of the disease rather than a later consequence of muscle necrosis [38,39,40,41,42,43]. Damaged or defective mitochondria can be evidenced by poor mitochondrial function (i.e., reduced ATP production), mitochondrial swelling/enlargement, lowered mitochondrial membrane potential, and the excessive release of ROS. In healthy tissue, these damaged mitochondria are marked for mitophagy, which refers to autophagy (or removal) of mitochondria, which mitigates the possible damage that can be caused by defective mitochondria-induced pathways. However, it has been shown that mitophagy is significantly impaired in DMD, as evidenced by high amounts of large, damaged mitochondria in the muscle, decreased mitophagy-related genes, and increased ROS levels. Thus, compounds such as resveratrol that suppress or reduce reactive oxygen species (ROS; highly reactive free radicals) levels have shown some benefit in reducing dystrophic symptoms in DMD mouse models and cells through the reduction of mitophagy [44,45,46,47]. 

Mitophagy is a process that is mediated by the PINK1 (PTEN-induced kinase 1)/Parkin (Parkinson juvenile disease protein 2, *PARK2*) pathway [48]. In healthy mitochondria, PINK1 will bind to the outer mitochondrial membrane but gets continuously degraded by the presenilin-associated rhomboid-like (PARL) protease of the inner mitochondrial membrane, resulting in the maintenance of the mitochondria (mitophagy signal stopped). However, in a damaged mitochondrion with lowered mitochondrial membrane potential, PINK1 does not get degraded and is able to signal to Parkin, an E3-ubquitin ligase, to tag the mitochondria for degradation. That consequently recruits p62, an autophagy adapter, which leads to the encapsulation of the mitochondria by LC3 autophagosomes which becomes subsequently degraded by lysosomes [49]. In both DMD patients and animal models of DMD (mouse and worms), critical mitophagy-related genes such as *PINK1*, *PARK2*, and *BNIP3*, were markedly decreased [39]. Interestingly, it was recently shown that DMD mRNA levels positively correlate with the expression of mitophagy genes. In Becker muscular dystrophy (BMD), which typically results from in-frame *DYSTROPHIN* variants resulting in partial dystrophin function, there is also impaired mitophagy (although not as severe as that resulting from the full dystrophin loss of the dystrophin protein in DMD) [6,39]. Moreover, it was shown that female *mdx* mice that carry one of the mutated DMD alleles also demonstrate mitochondrial dysfunction and impaired mitophagy [38]. Taken together, this highlights that even partial loss of dystrophin is sufficient to induce mitochondrial dysfunction and impaired mitophagy, and that complete loss of dystrophin as seen in DMD has profound effects on mitophagy in muscle. 

Impaired mitophagy is important in DMD pathology as it results in the accumulation of damaged or defective mitochondria which release high amounts of ROS, mtDNA, and cardiolipin, thus increasing the oxidative stress of the cell [46]. This is clearly demonstrated in animal models where knockout of one of the key mitophagy genes, such as *Atg5* or *Pink1*, results in increased ROS levels. Ultimately, the large amounts of dysfunctional mitochondria contribute to muscle damage with increased ROS and by decreasing the overall available energetics of the cell (reduced overall ATP production due to impaired mitochondrial respiration). Impaired mitophagy was not limited to skeletal muscle, as previous work reports it is also present in dystrophic cardiomyopathy [50], which worsened with disease progression. 

Another aspect to consider is the excessive calcium overload in the cells. Due to membrane fragility resulting from the loss of dystrophin, increased calcium enters the cell without regulation. High intracellular calcium can cause several damaging downstream cascades, one of which is involved with mitochondria. Alongside the endoplasmic/sarcoplasmic reticulum, mitochondria are large calcium sinks in the cell. Under abnormally high intracellular calcium concentrations, however, the calcium overloading in mitochondria cause mitochondrial swelling and ROS production [40]. Additionally, this excess calcium results in the breakdown of mitochondrial structure (as seen by the decreased loss of cristae) by irreversibly opening the mitochondrial permeability transition pore (mPTP), as mitochondrial structure loss was correlated with increased mitochondrial calcium levels [50]. ROS species are primarily derived from mitochondria, although they can also result from NADPH oxidases, lipoxygenases, or other sources. It is important to note that ROS species are not inherently bad as they also serve an important physiological role and act as a secondary messenger to report a status of ATP availability and adaptation to stress [51]. There are endogenous free radical scavengers, such as antioxidants, that tightly regulate ROS species and render them safe and non-damaging by reducing them. However, if this system becomes dysregulated and there is a significant and chronic imbalance that results in high ROS levels, that is when oxidative stress occurs [51]. 

Importantly, therapeutic approaches that increase mitophagy with a concomitant reduction in ROS levels have been shown to be effective in ameliorating DMD pathological outcomes. There are different ways to target this mechanism, including treating with antioxidants [52,53], activating the AMP kinase (AMPK) pathway [54,55,56], increasing NAD^+^-dependent Sirtuin 1 (SIRT1)/peroxisome proliferator-activated receptor gamma coactivator (PGC-1α) pathway [44,46,57,58,59,60], and upregulating mitophagy-related genes [39]. Many antioxidants have shown the therapeutic efficacy in DMD mice as seen by improvement of DMD muscle outcomes, either by acting as an ROS scavenger or by preventing the ROS formation [51]. Alternatively, there are therapeutic approaches that indirectly can also improve mitophagy and mitochondrial function, including calcium modulators [61]. 

### 1.4. Mitochondria and Inflammation

Earlier we discussed two main inflammatory pathways that are upregulated in DMD pathology: the NFκB pathway and the NLRP3 inflammasome pathway. Recent evidence indicates that mitochondria may play a central role in regulating these pathways as there is a lot of cross-talk between the mitochondria and the immune response [62]. AMPK is an energy sensor of the cell. It has the ability to stimulate mitochondrial biogenesis via PGC-1α, or induce the breakdown of damaged mitochondria via mitophagy [56,62,63]. Activated p-AMPK can induce the removal of damaged mitochondria through general autophagy pathways involving ULK1, ATG3 and ATG12 [62,63], and its loss can also result in defects in mitophagy [63]. AMPK also plays a significant role in inflammation. It was recently shown that AMPK activation can result in the reduction of latent-TGFβ1 by decreasing *LTBP4* expression in macrophages. Additionally, AMPKα1-deficient macrophages were unable to transition from M1 pro-inflammatory macrophage phenotype to M2 pro-regenerative macrophage phenotype, which is critical for proper tissue regeneration [64].

SIRT1 is a NAD^+^-dependent deacetylase localized to the mitochondria, which can deacetylase PGC-1α to turn on its co-transcriptional activity and promote mitochondrial biogenesis [65]. In addition, SIRT1 can also deacetylase NFκB at subunit p65, which renders NFκB non-active and thus reduces pro-inflammatory gene transcription. Additionally, PGC-1α can regulate NFκB signaling by reducing the phosphorylation of NFκB member p65, thus blocking its transcriptional activity of inflammatory cytokines [66]. Moreover, PGC-1α was also shown to increase the expression of anti-inflammatory cytokines and may help in skewing macrophages to the M2 phenotype, which is associated with pro-regenerative mechanisms [67]. However, NFκB activation can also result in the reduction of SIRT1 and PGC-1α expression, diminishing the oxidative metabolism of the cell and promoting inflammation [68,69,70]. This highlights the complex relationship between mitochondrial components and inflammation mechanisms. 

While healthy mitochondria can regulate parts of the immune response and reduce inflammatory signals, damaged mitochondria lead to the activation of both the NFκB pathway and the NLRP3 inflammasome [71]. ROS activates the NFκB pathway, which in turn leads to the upregulation of pro-inflammatory cytokines such as TNFα and interleukin (IL)-1β. Interestingly, there may be the potential of a positive inflammatory feedback loop as TNFα can increase mitochondrial ROS levels, thereby reinforcing the redox imbalance and inflammatory pathway [47]. Moreover, damaged mitochondria can activate the NLRP3 inflammasome with increased ROS and cardiolipin release [71]. Interestingly, cells treated with NLRP3 inflammasome agonists show increased mitochondrial damage and reduced mitochondrial membrane potential, yet this damage was shown to be independent of direct NLRP3 inflammasome components. This suggests that mitochondrial damage is upstream of NLRP3 and acts to activate the NLRP3 inflammasome [72]. Furthermore, the mitochondrial damage mediated by NLRP3 agonists are enhanced under conditions with lowered mitophagy, such as when any of the critical mitophagy genes are ablated [72]. One example of this was seen when NLRP3 agonists were treated to *Park2*-deficient cells, which led to increased IL-1β and caspase-1 activity [72]. Taken together, these data demonstrate that NLRP3 agonists can cause accumulation of damaged mitochondria, and impaired mitophagy of these mitochondria result in the promotion of pro-inflammatory pathways. Thus, therapeutic targets that either upregulate mitophagy or reduce downstream cascades induced by damaged mitochondria (ROS, mtDNA, cardiolipin) may also improve inflammation. This would be of great interest in DMD, as both mitophagy and inflammation are greatly affected. 

### 1.5. Possible Therapeutic Targets for Improving Mitophagy and Inflammation in DMD

We have highlighted the relationship between mitochondria and inflammation, and how impaired mitophagy leads to increased inflammation (Figure 1). As mitophagy is impaired and chronic inflammation is present in DMD, this represents a therapeutic approach that may be beneficial in attenuating DMD disease pathology severity. Additionally, studies have shown that treatments that aim to reduce the secondary downstream effects of damaged mitochondria (increased ROS and cardiolipin) are sufficient at reducing DMD disease pathology. For example, studies have shown that treatments with compounds that target either ROS or mtDNA in impaired mitophagy conditions (such as p62 or *Park2* deficiency) are still effective in reducing NLRP3 inflammasome effects and reduced the release of IL-1β [72]. AMPK activation via the treatment with AMPK agonist AICAR in *mdx* mice improved mitophagy, reduced ROS levels and upregulated PGC-1α and utrophin expression, resulting in the improvement of muscle function and histological pathology markers [56,59,73]. Consistent with this, treatment with a different synthetic agonist of AMPK, metformin, in *mdx* mice also resulted in an amelioration of dystrophic pathology by the upregulation of PGC-1α and utrophin expression [74]. Furthermore, a recent study demonstrated that metformin treatment was also able to reduce the detrimental SR/ER-mitochondrial interaction seen in dystrophic cardiomyocytes and restore mitochondrial function [55]. 

There have been many different compounds that have been found to activate the SIRT1/PGC-1α axis in *mdx* mice (recently reviewed by Suntar et al. [60]). One of these compounds is resveratrol, a natural compound that increases SIRT1 expression which stimulates the SIRT1/PGC-1α axis in *mdx* mice [75,76]. Several studies have shown the efficacy of resveratrol treatment in ameliorating dystrophic pathology in *mdx* mice, showing it can improve mitophagy [44,46], reduce ROS levels [76], reduce inflammation [45,77,78], and increase utrophin [77] expression. One study compared the beneficial effects of resveratrol to the gold standard α-methyl prednisolone. Both treatments increased in vivo muscle function, reduced plasma creatine kinase levels, and improved muscle histology [79], suggesting that resveratrol and other compounds that target the SIRT1/PGC-1α pathway are strong candidate targets for therapeutics of DMD. Antioxidants are the natural defense against excessive and damaging ROS, where they can render the ROS to be neutral. As ROS levels are excessive in DMD, there have been numerous studies measuring the therapeutic benefit of different types of antioxidants in DMD [80,81,82,83,84]. One example is the synthetic antioxidant Tempol (4-hydroxy-2,2,6,6-tetramethylpiperidine-N-oxyl) that acts similarly to superoxide dismutase (SOD), which was investigated as a therapeutic in DMD pathology. Tempol treatment improved the dystrophic phenotype as seen by increases in muscle force, reducing inflammation, reducing oxidative stress, and decreasing muscle necrosis [52,85,86,87]. Interestingly, there is recent evidence that deflazacort, a steroid commonly prescribed for DMD patients, is able to restore mitochondrial function [88]. Deflazacort treatment in *mdx* mice resulted in the upregulation of mitochondrial complex proteins as well as a significant decreases of mitochondrial calcium importation [88]. This is associated with increased ATP energetics and likely contributes to the overall therapeutic benefits seen with the anti-inflammatory effects of deflazacort. 

## 2. Conclusions

It is evident that mitophagy and inflammation play a critical role in DMD pathology and that therapeutic approaches that target this pathway ameliorates DMD disease outcomes. While there is promising evidence to help attenuate DMD disease pathology, most of these studies have been limited to DMD animal models and only few have progressed into clinical trials in DMD patients (recently reviewed in Verhaart et al. [89]). Interestingly, it has been shown that treatment with NFκB inhibitors allowed for a higher mini-dystrophin expression induced via AAV treatment in *mdx* mice than without treatment, suggesting that it helps the transduction of the mini-dystrophin into the muscle [90]. This supports the idea of a combinatorial therapy for DMD that will involve anti-inflammatory/antioxidant treatment with a gene therapy to restore muscle stability and function. However, further research is necessary to understand the roles of the aforementioned candidate therapeutics in DMD patients already on glucocorticoid regimens, as there could be possible safety issues or pharmacological interactions [91]. Until these interactions are fully elucidated, patients should refrain from self-supplementing with therapeutics that have not been discussed with their physician. Nevertheless, there is optimism that by understanding the mechanistic links between mitophagy, inflammation, and dystrophinopathy, disease processes and more targeted combinatorial approaches can be designed to treat this devastating disease.

## Data Availability

Not applicable.
